# Biochemical and histopathological changes related to the topical application of *Aloe vera* ointment for canine pyoderma

**DOI:** 10.14202/vetworld.2021.1354-1362

**Published:** 2021-05-28

**Authors:** Ali Arbaga, Amanallah El-Bahrawy, Ahmed Elsify, Hadeer Khaled, Hany Youssef Hassan, Ahmed Kamr

**Affiliations:** 1 Department of Animal Medicine and Infectious Diseases, Faculty of Veterinary Medicine, University of Sadat City, Sadat City, Egypt; 2 Department of Veterinary Pathology, Faculty of Veterinary Medicine, University of Sadat City, Sadat City, Egypt

**Keywords:** *Aloe vera* biochemical and histopathology examination, gentamicin, pyoderma, *Staphylococcus aureus*

## Abstract

**Background and Aim:**

Pyoderma is common in dogs, and its treatment requires a novel medication rather than antibiotic therapy. This study aimed to determine the biochemical and histopathological changes associated with the topical application of *Aloe vera* 20% and 40% ointments, compared with gentamicin 0.1% ointment, in dogs suffering from *Staphylococcus aureus* pyoderma.

**Materials and Methods:**

Serum and skin samples were collected from a negative control group before inducing pyoderma and from other subdivided groups on the 3^rd^, 7^th^, 10^th^, and 14^th^ days post-inoculation for biochemical and histopathology examination.

**Results:**

Serum aspartate aminotransferase, alanine aminotransferase (ALT), urea, and creatinine concentrations were higher in the positive control dogs on the 3^rd^ day without treatment (DWT) compared with the negative control dogs (p<0.05). Compared with the healthy control dogs, serum zinc concentrations were lower in the positive control group on the 3^rd^, 7^th^, and 10^th^ DWT and in dogs treated with *A. vera* 20% and gentamicin 0.1% ointments on the 3^rd^ and 7^th^ days post-treatment (p<0.05). Grossly, skin had erythema, pruritus, and pus-filled pustules of the untreated group. Microscopically, skin showed epidermal necrosis and edema, dermal collagen necrosis, and severe neutrophilic infiltration.

**Conclusion:**

Compared with *A. vera* 20% and gentamicin 0.1% ointments, the topical application of *A. vera* 40% ointment-induced quicker skin healing and decreased the inflammatory changes caused by *S. aureus* inoculation, based on biochemical and histopathological changes reflective of its curative efficiency. *A. vera* 40% ointment may be a suitable alternative to antibiotics for the treatment of staphylococcal pyoderma in dogs.

## Introduction

*Aloe vera* belongs to the *Asphodelaceae* (*Liliaceae*) family. It is naturally cultivated throughout Africa, Asia, Europe, and America in tropical or subtropical regions. *A. vera* is registered as a medicinal drug of herbal origin in Egypt, India, Greece, Rome, and China [1]. *A. vera* extract consists of two primary parts: Latex and gel. *A. vera* gel is composed of 98.5-99.5% water, and the remaining dry matter contains more than 75 biologically active ingredients [2,3], which are helpful for the treatment of diseases. Major *A. vera* components include anthraquinones, polysaccharides, vitamins, enzymes, and low-pollutant components [4]; because of these components, *A. vera* has anti-inflammatory, immunomodulatory, wound healing, antiviral, antifungal, antitumor, antidiabetic, and antioxidant properties [5].

Canine pyoderma is a common disease, and staphylococcal folliculitis is the most frequently observed type in dogs [6-8]. Canine pyoderma is mainly caused by *S. intermedius* [6]; however, up to 10% of cases can be caused by *Staphylococcus aureus* and a recent emergent strain *Staphylococcus schleiferi* [6,8]. Antibiotic therapy, either locally applied or injected, is the usual protocol for treating staphylococcal pyoderma [6,7,9,10]. *S. aureus* can acquire resistant genes and overcome the inhibitory effects of antibiotics through several resistance mechanisms [11]. However, the potential role of *A. vera* in the treatment of canine staphylococcal pyoderma requires further investigation.

Several antibiotics are traditionally used to treat *S. aureus*, one of which is aminoglycoside gentamicin [11]. Gentamicin used to treat *S. aureus* infections by binding with the 30S ribosomal subunit and inhibiting bacterial protein synthesis; its efficiency has decreased as the bacteria have acquired resistance encoded by mobile genetic elements [12]; therefore, treatment with gentamicin has begun to have a low value. Thus, there is a need to find an additional product for the treatment of staphylococcal pyoderma, particularly if this product is cheap, readily available, and of a natural source.

This study aimed to evaluate the biochemical and histopathological changes related to the topical application of *A. vera* ointment as a potential new treatment and compared its effects to those of a traditional treatment (gentamicin ointment) in dogs suffering from pyoderma. We hypothesized that, as a medicinal plant, *A. vera* would have a beneficial effect compared with gentamicin in treating staphylococcal pyoderma in dogs based on biochemical and histopathological assessments.

## Materials and Methods

### Ethical approval

All procedures in this study were approved by the University of Sadat City for the Care and Use of Animals in Education and Scientific Research (Approval code VUSC-007-1-19).

### Study period and location

This study was carried out from September 2018 to May 2019. The experiment was carried out at the Department of Animal Medicine and Infectious Diseases, University of Sadat City.

### Animals

Twenty 2-3-year-old male dogs were housed for 2 weeks to acclimatize to individual cages (120 cm×140 cm×160 cm (width×height×depth)) and had free access to food and water. Ivermectin 1% [(Paramectin^®^,Pharma Swede, Egypt] was used for deworming by SC injection at a dosage of 10 mg/50 kg in compliance with manufacturer’s guidelines [13].

### Experimental design

Each dog’s fur was shaved, and the skin was washed with sterile water and soap. Under local anesthesia, dogs were intradermally inoculated with 1 ml broth containing 10^5^ colony-forming unit of *S. aureus RMSA4* strain as previously described [13,14]. Three days after inoculation, skin pyoderma appeared. To confirm the identity of the observed skin lesions, on the 3^rd^ day post-inoculation, lesions were swapped with a sterile swab. Samples were subjected to gram staining and cultured on Baird–Parker agar medium at 37°C for 24 h for the identification of the grown colonies. Dogs were further divided into positive control untreated group (n=5) and treated groups with *A. vera* ointment at 20% (n=5) and 40% (n=5) and gentamicin 0.1% ointment (n=5). *A. vera* 20% and 40% ointments were prepared by our research group [13,15], whereas the gentamicin sulfate 0.1% ointment was commercially purchased (Garamyci^n®^ ointment 0.1%, Schering-Plough Company, USA).

Topical treatment was performed twice daily with 1 g of *A. vera* 20% and 40% and gentamicin 0.1% ointments until complete skin healing. Blood serum samples were collected for the colorimetric determination of aspartate aminotransferase (AST), alanine aminotransferase (ALT), blood urea nitrogen (BUN), creatinine, zinc, and glucose concentrations. The skin was grossly examined and scored from 0 to 4 according to the progression of healing. A score of 0 corresponded to the presence of erythema, pruritus, and pustules filled with a large amount of pus. A score of 1 corresponded to pruritus and pustules filled with a moderate amount of pus. A score of 2 corresponded to pustules with a small amount of pus. A score of 3 corresponded to the absence of pus and epidermal collarette. A score of 4 corresponded to complete healing.

### *S. aureus* culture

The *S. aureus RMSA4* strain used in this study was provided by the Department of Food Hygiene, Faculty of Veterinary Medicine, University of Sadat City, Egypt. This strain possessed virulence factors such as coagulase-positive and genes encoding virulence. In addition to biochemical identification, this strain was confirmed to be *S. aureus* by polymerase chain reaction, as previously described [16]. This strain was sensitive to cefoxitin, gentamicin, kanamycin, erythromycin, tetracycline, doxycycline, ciprofloxacin, chloramphenicol, trimethoprim-sulfamethoxazole, and penicillin.

### Re-isolation and identification of the inoculated staphylococcus strain from dog lesions

Swabs were collected from the lesion after its appearance. Each swab was directly cultured on a plate with Baird–Parker agar medium at 37°C for 24 h. The grown colonies were identified as previously described [17].

### Serum AST, ALT, BUN, creatinine, glucose, and zinc concentrations

We assessed and determined the AST and ALT levels using a liver function test, BUN and creatinine levels by a kidney function test, and glucose and zinc levels by colorimetric methods using special kits (Bio-Diagnostic Company, Giza, Egypt).

### Tissue sampling and histopathology procedures

Punch biopsies (5 mm) were collected on day 0 (the 3^rd^ day after the appearance of pyoderma) and on the 3^rd^, 7^th^, 10^th^, and 14^th^ days post-treatment (DPT). Biopsy samples were preserved for 3 days in 10% neutral buffered formalin and then routinely processed and embedded in paraffin blocks. Paraffin tissue sections (4 mm) were cut, dried, and stained with hematoxylin and eosin stain for examination under a light microscope [18]. Sections were semiquantitatively scored as follows: –, none; +, mild < 25%; ++, moderate < 50%; and +++, severe > 50% of examined sections. Six sections were examined and counted from the skin of each dog.

### Statistical analysis

Data normality was assessed using a Shapiro–Wilk test; data were normally distributed and expressed as mean with standard error. A one-way analysis of variance was used to compare differences between the groups using IBM SPSS statistics version 16 (IBM Corporation, NY, USA) [19]. Significance was considered at p<0.05.

## Results

### Isolation and identification of *S. aureus* bacteria from dog lesions

**Figure-1:**
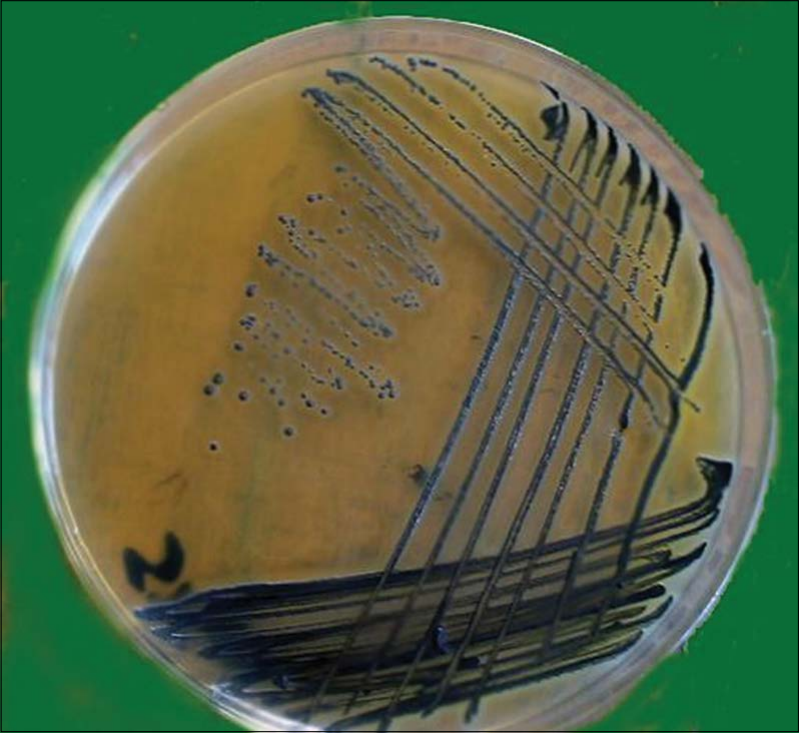
*Staphylococcus aureus* on Baired-Parker medium showed black, convex, shiny, colonies, and 1-1.5 mm in diameter surrounded by clear zone.

The inoculated strain was confirmed through lesion swab cultures on Baird–Parker agar. Growing colonies were black, convex, and shiny and measured 1-1.5 mm in diameter with clear margins, which are characteristic of *S*. *aureus* (Figure-1). In addition, grapelike Gram-positive cocci appeared on Gram-stained smears under the microscope. The isolated strain showed the biochemical reactions, which are characteristic of *S*. *aureus*, as shown in Table-1.

**Table-1:** Biochemical reactions of isolated *S. aureus* from dogs’ pyoderma.

Biochemical test	Isolated strain
Oxidase	−
Catalase	+
Coagulase	+
DNase	+
Hemolysis	+
Pigment production	+
Alkaline phosphatase	+
Urease	+
Mannitol	+
Maltose fermentation test	+
Novobiocin 5mcg	Sensitive
Polymix n B 300- unit disc	Resistant

*S. aureus=Staphylococcus aureus*

### Serum AST, ALT, BUN, creatinine, glucose, and zinc concentrations

Biochemical profiles revealed elevated serum AST and ALT concentrations in infected dogs on the 3^rd^ day without treatment (DWT) compared with negative control dogs (p<0.05); however, there were no differences in the AST and ALT concentrations of dogs treated with *A. vera* 20% and 40% and gentamicin 0.1% at all time points compared with negative control dogs (p>0.05; Table-2). Serum BUN and creatinine concentrations were higher in the positive control group on the 3^rd^ and 7^th^ DWT and on the 3^rd^ DPT in dogs treated with *A. vera* 20% and gentamicin 0.1% compared with negative control dogs (p<0.05). There were no statistical differences in urea and creatinine values between dogs treated with *A. vera* 20% and 40% and gentamicin 0.1% ointments on the 7^th^, 10^th^, and 14^th^ DPT compared with negative control dogs (p>0.05; Table-2). Serum zinc concentrations were lower in the positive control group on the 3^rd^, 7^th^, and 10^th^ DWT and in dogs treated with *A. vera* 20% and gentamicin 0.1% ointments on the 3^rd^ and 7^th^ DPT compared with healthy control dogs (p<0.05); however, they did not differ from those of dogs treated with *A. vera* ointment 40% (p>0.05). Serum glucose concentrations were only lower in untreated infected dogs on the 3^rd^ and 7^th^ DWT. Serum glucose concentrations also decreased in dogs treated with *A. vera* 20% ointment on the 3^rd^ DPT compared with negative control dogs (p<0.05), but these concentrations were no different from those of dogs treated with *A. vera* 40% and gentamicin 0.1% ointments (p>0.05; Table-2).

**Table-2:** Biochemical profile of experimentally induced pyoderma in dogs treated by *Aloe vera* 20%; 40%; and gentamicin 0.1% ointments.

Variables	ALT (U/L)	AST (U/L)	Urea (mmol/l)	Creatinine (mg/dl)	Zinc (μmol/L)	Glucose (mmol/L)
Control negative (before induction) (n=20)	34.6±1.82^a^	36.8±0.8^a^	7.28±0.6^a^	0.82±0.3^a^	18.2±1.2^a^	4.8±0.4^a^
Infected dogs without treatment (n=5)					
3^rd^ DWT	46.28±0.9^b^	48.7±2.2^b^	12.2±0.8^b^	1.4±0.6^b^	11.8±0.6^b^	2.8±0.4^b^
7^th^ DWT	35.5±0.8^a^	38.2±1.2^a^	11.6±0.4^b^	1.2±0.78^b^	12.7±0.8^b^	3.2±1^b^
10^th^ DWT	34.8±1.08^a^	36.4±2.08^a^	8.6±0.6^a^	0.88±0.5^a^	12.6±1.1^b^	4.2±0.8^a^
14^th^ DWT	36.04±0.5^a^	36.68±1.3^a^	8.2±0.6^a^	0.82±0.4^a^	16.8±0.6^a^	4.6±0.6^a^
Dogs treated with *Aloe vera* gel ointment 20% (n=5)					
3^rd^ DPT	37.4±0.68^a^	36.2±1.4^a^	8.2±0.58^b^	0.84±0.8^a^	12.2±1.2^b^	4.2±0.4^b^
7^th^ DPT	34.8±1.1^a^	38.4±0.8^a^	8.08±0.8^a^	0.82±0.3^a^	12.8±0.5^b^	4±0.5^a^
10^th^ DPT	36.2±0.8^a^	36.8±1.1^a^	7.2±1.06^a^	0.88±0.4^a^	16.8±0.8^a^	4.5±0.3^a^
14^th^ DPT	34.12±0.8^a^	37.2±0.6^a^	8±0.4^a^	0.78±0.6^a^	18.06±0.6^a^	4.18±0.4^a^
Dogs treated with *A. vera* gel ointment 40% (n=5)					
3^rd^ DPT	36.08±0.4^a^	36.8±1.18^a^	8.4±0.4^a^	0.85±0.2^a^	13.2±0.8^b^	4.3±0.7^a^
7^th^ DPT	36.4±0.38^a^	37.08±0.6^a^	8.7±1.02^a^	0.82±0.4^d^	17.4±1.2^ a^	4.8±0.3^a^
10^th^ DPT	34.2±0.8^a^	35.8±0.8^a^	8.02±0.54^a^	0.88±0.6^a^	18.2±0.6^a^	4.2±0.6^a^
14^th^ DPT	34.7±0.6^a^	36.6±1.04^a^	7.6±0.4^a^	0.80±0.3^a^	18.4±0.6^a^	4.6±0.4^a^
Dogs treated by gentamicin sulfate ointment 0.1% (n=5)					
3^rd^ DPT	36.12±0.4^a^	35.8±0.4^a^	7.85±0.4^b^	0.88±0.4^c^	12.28±0.6^b^	4.2.8±0.6^a^
7^th^ DPT	34.4±0.68^a^	36.8±0.6^a^	8.06±0.5^a^	0.84±0.2^d^	13.2±0.8^b^	4.8±0.8^a^
10^th^ DPT	35.42±0.5^a^	37.2±0.8^a^	8±0.68^c^	0.78±0.5^a^	17.2±0.5^a^	4.6±0.2^a^
14^th^ DPT	34.8±0.62^a^	36.2±1.2^a^	8.2±0.4^c^	0.82±0.4^a^	18±1.06^a^	4.2±0.7^a^

n=Number. Means with different letter superscripts in the same column are significantly different at (p<0.05). *A. vera*=*Aloe vera*, ALT=Alanine aminotransferase, AST=Aspartate aminotransferase, DWT=Day without treatment, DPT=Days post-treatment

### Gross examination

**Figure-2:**
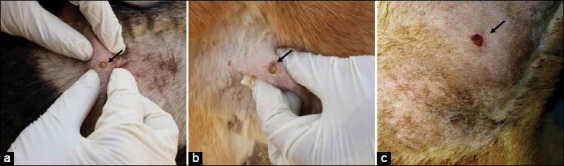
Untreated group. (a) Zero day of the experiment, the skin showing erythema, pruritis, and pus-filled pustules (arrow). (b) 10^th^ day without treatment (DWT), the skin showing lesion with hyperemic rim, erythema and pus center (arrow). (c) 14^th^ DWT, the skin showing epidermal collarette the start of scar formation over the lesion (arrow).

**Figure 3:**
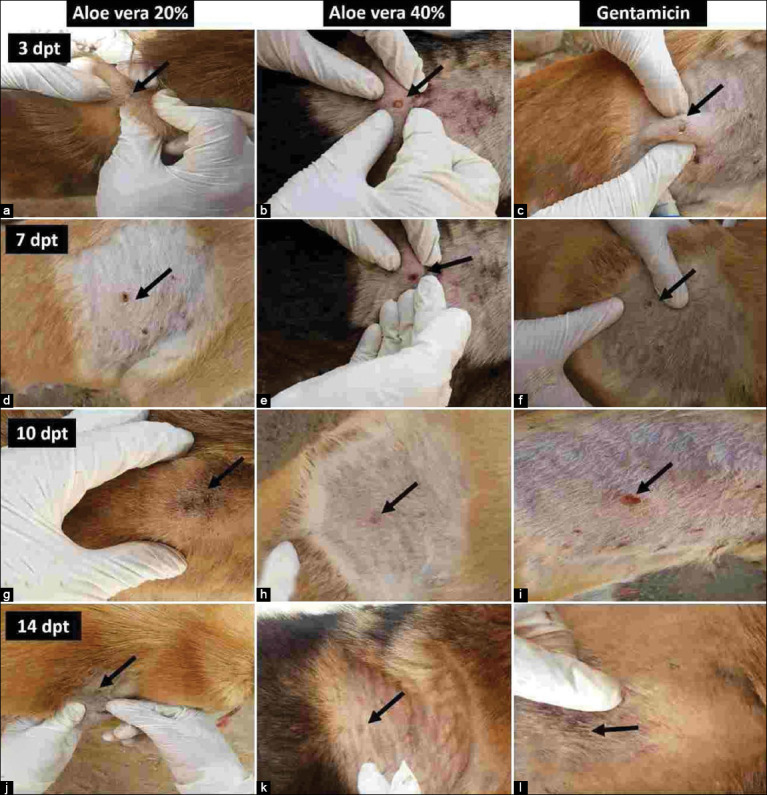
Treated group. On 3^rd^ days post-treatment (DPT), (a) Skin treated with *Aloe vera* ointment 20% showed erythema, pus filled pustules. (b) Skin treated with *A. vera* ointment 40% showed erythema, pruritis, and presence of pus. (c) Skin treated with gentamicin 0.1% showed erythema and whitish pus content. On 7^th^ DPT, (d) skin treated with *A. vera* ointment 20% showed erythema with moderate pus content. (e) Skin treated with *A. vera* ointment 40% showed erythema with less pus content. (f) Skin treated with gentamicin ointment 0.1% showed erythema and moderate pus content. On 10^th^ DPT, (g) skin treated with *A. vera* ointment 20% showed small amounts of pus. (h) Skin treated with *A. vera* ointment 40% showed epidermal collarette with absence of pus. (i) Skin treated with gentamicin ointment 0.1% showed little pus. On 14^th^ DPT, (j) skin treated with *A. vera* ointment 20% showed epidermal collarette with absence of pus. (k) Skin treated with *A. vera* ointment 40% showed complete healing. (l) Skin treated with gentamicin ointment 0.1% showed epidermal collarette. Lesions are indicated by arrows.

Compared with the positive control, treatment with *A. vera* 40% ointment induced faster healing than treatment with *A. vera* 20% and gentamicin 0.1% ointments; the healing effect of *A. vera* 20% ointment was equal to that of gentamicin 0.1%. The lesion scores for the untreated and treated groups are summarized in Table-3. Grossly, skin displayed signs of inflammation, such as erythema, pruritus, and pus-filled pustules. On day 0 of the experiment, the skin of all inoculated dogs was hyperemic, painful, and pruritic with excessive whitish thick pus-filled pustules (Figure-2a). On the 3^rd^ DWT, dogs in the untreated group had severe forms of the aforementioned signs; whereas dogs treated with *A. vera* 20% and 40% and gentamicin 0.1% ointments had moderate pruritus and a moderate amount of pus (Figures-3a-c). On the 7^th^ DWT, dogs in the untreated group continued to have these severe signs, whereas dogs treated with *A. vera* 20% and gentamicin 0.1% ointments also had moderate pruritus and a moderate amount of pus; on the other hand, dogs treated with *A. vera* 40% ointment had only few amount of pus on 7^th^ DPT (Figures-3d-f). On the 10^th^ DWT, untreated dogs had moderate pruritus and a moderate amount of pus (Figure-2b), whereas dogs treated with *A. vera* 20% and gentamicin 0.1% ointments had small amounts of pus, and dogs treated with *A. vera* ointment 40% showed epidermal collarette and an absence of pus (Figures-3g-i). On the 14^th^ DWT, untreated dogs had moderate pruritus, a moderate amount of pus, and the beginning of scar formation (Figure-2c), whereas dogs treated with *A. vera* 20% and gentamicin 0.1% ointments had epidermal collarette and an absence of pus, and dogs treated with *A. vera* ointment 40% showed complete healing and a complete absence of inflammatory signs (Figures-3j-l).

**Table-3:** Gross lesions score of experimental infection of dog skin with *S. aureus* and topical treatment with *A. vera* 20%, 40%, and gentamicin 0.1% ointments.

Time	Gross lesions score			

	Untreated group	*A. vera* 20% ointment	*A. vera* 40% ointment	Gentamicin 0.1% ointment
Zero day	0	0	0	0
3^rd^ DPT	0	1	1	1
7^th^ DPT	0	1	2	1
10^th^ DPT	1	2	3	2
14^th^ DPT	1	3	4	3

DPT=Days post pyoderma treatment, Lesions score: 0=Erythema, severe pruritis and pustules filled with much pus, 1=Moderate pruritis and pustules filled with moderate amount of pus, 2=Mild pruritis and pustules with little pus, 3=Absence of pus and epidermal collarette, 4=Complete healing. *A. vera*=*Aloe vera*, *S. aureus=Staphylococcus aureus*, DPT=Days post-treatment

### Histopathological examination

**Figure-4:**
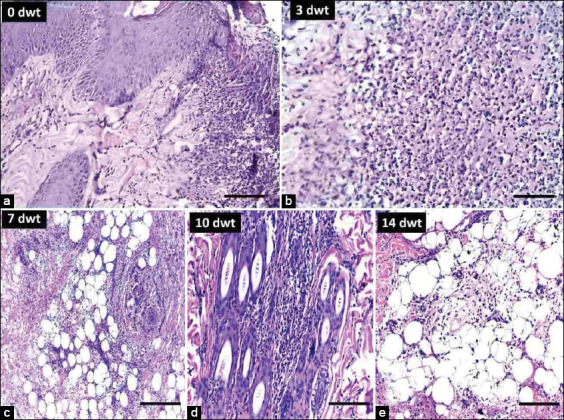
Untreated group. (a) On zero day of the experiment, the skin showing epidermal necrosis dermal collagen necrosis and severe neutrophils infiltration in the epidermis, dermis, and around the hair follicles; H and E 20×. (b) On 3^rd^ day without treatment (DWT), the skin showed severe necrosis and neutrophil infiltration; H and E 40×. (c) On7^th^ DWT, the skin has severe inflammation in the subcutaneous fat and arteritis; H and E 10×. (d) On 10^th^ DWT, the skin showing inflammatory cells invades the hair follicles; H and E 20×. (e) On 14^th^ DWT, the skin showing moderate inflammatory cells in the subcutaneous tissue; H and E 20×.

**Figure-5:**
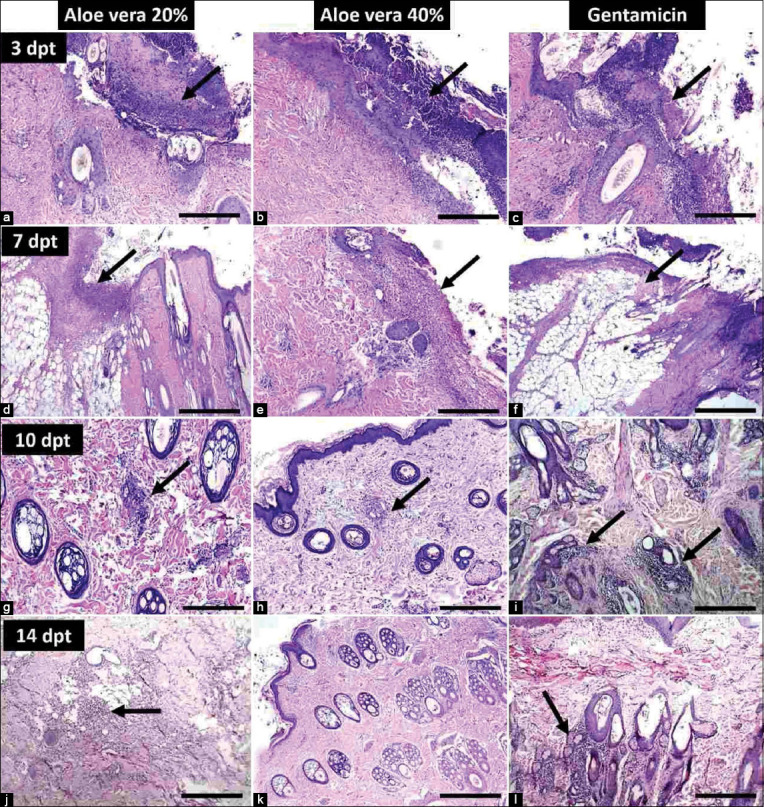
Treated group. On 3^rd^ days post-treatment (DPT), (a) skin treated with *Aloe vera* ointment 20% showed epidermal necrosis, edema, severe neutrophilic infiltration, and hair folliculitis; H and E 10×. (b) Skin treated with *A. vera* ointment 40% showed epidermal necrosis, edema, and moderate neutrophilic infiltration; H and E 10×. (c) Skin treated with gentamicin ointment 0.1% showed epidermal necrosis, edema, severe neutrophilic infiltration, and hair folliculitis; H and E 10×. On 7^th^ DPT, (d) Skin treated with *A. vera* ointment 20% showed focal suppurative inflammation in the subcutaneous fat; H and E 10×. (e) Skin treated with *A. vera* ointment 40% showed epidermal necrosis with few neutrophils’ infiltration; H and E 4×. (f) Skin treated with gentamicin ointment 0.1% showed diffuse suppurative inflammation in the epidermis and subcutaneous fat; H and E 4×. On 10^th^ DPT, (g) skin treated with *A. vera* ointment 20% showed mild inflammation in the dermis and around the hair follicles; H and E 10×. (h) Skin treated with *A. vera* ointment 40% showed mild inflammatory zone in the dermis; H and E 10×. (i) Skin treated with gentamicin ointment 0.1% showed mild inflammation around the hair follicles; H and E 10×. On 14^th^ DPT, (j) skin treated with *A. vera* ointment 20% showed mild inflammatory cells in the subcutaneous tissue; H and E 4×. (k) Skin treated with *A. vera* ointment 40% showed normal histological architecture of skin; H and E 4×. (l) Skin treated with gentamicin ointment 0.1% showed mild folliculitis; H and E 4×. Lesions are indicated by arrows.

Microscopically, the topical application of *A. vera* 40% ointment induced faster skin healing and decreased the inflammatory changes caused by *S. aureus* inoculation than *A. vera* 20% and gentamicin 0.1% ointment. The results are summarized in Table-4. On day 0 of the experiment (the 3^rd^ day after the appearance of lesions), skin showed epidermal necrosis and edema, dermal collagen necrosis, and severe inflammatory cells infiltration, mainly with neutrophils in the epidermis, dermis, and around the hair follicles (Figure-4a). Inflammation was deeply extended to involve subcutaneous fat and blood vessels. These neutrophilic infiltrations were present as either aggregate or in a diffuse manner in both the epidermis and dermis. On the 3^rd^ DWT, the epidermis and dermis of untreated dogs had severe necrosis, edema, and neutrophilic infiltration diffusely observed around hair follicles and blood vessels (Figure-4b). On the 3^rd^ DWT, dogs topically treated with *A. vera* 20% ointment and gentamicin 0.1% had moderate inflammatory changes in the epidermis and severe inflammatory changes in the dermis and subcutaneous tissue, whereas dogs topically treated with *A. vera* 40% ointment had moderate inflammatory changes throughout all skin layers (Figure-5c). On the 7^th^ DWT, inflammatory changes, folliculitis, and subcutaneous blood vessel inflammation were severe in the skin of dogs from the untreated group (Figure-4c). On the 7^th^ DWT, dogs topically treated with *A. vera* 20% ointment and gentamicin 0.1% showed moderate necrosis, infiltration of neutrophils, and few lymphocytes in the epidermis and dermis, whereas these changes were severe in the subcutaneous tissue, including blood vessels. On the 7^th^ DWT, dogs topically treated with *A. vera* 40% ointment showed mild neutrophilic infiltration that was focal in the epidermis and dermis and moderate neutrophilic infiltration that was diffuse in the subcutaneous tissue (Figures-5d-f). On 10^th^ DWT, inflammatory changes, including necrosis, neutrophilic infiltration, folliculitis, and subcutaneous fat and blood vessel inflammation, were severe and diffuse throughout all skin of dogs in the untreated group (Figure-4d). On the 10^th^ DPT, the epidermis and dermis of dogs topically treated with *A. vera* 20% and gentamicin 0.1% ointments demonstrated mild neutrophilic infiltration, whereas the subcutaneous tissue showed moderate inflammation and necrosis. On the 10^th^ DPT, dogs topically treated with *A. vera* 40% ointment showed an absence of inflammation in the epidermis, whereas inflammation was mild in the dermis and subcutaneous tissue (Figures-5g-i). On the 14^th^ DWT, in the untreated group, inflammatory changes, including necrosis and neutrophilic infiltration were moderate in the epidermis, dermis, and subcutaneous tissue (Figure-4e). On the 14^th^ DPT, the epidermis and dermis of dogs topically treated with *A. vera* 20% ointment demonstrated no inflammation, whereas the subcutaneous tissue showed mild inflammation, and the epidermis of dogs topically treated with gentamicin 0.1% had no inflammation, whereas the dermis and subcutaneous tissue showed mild inflammation. Finally, on the 14^th^ DPT, dogs topically treated with *A. vera* 40% ointment had complete skin healing and an absence of inflammatory signs in all skin layers (Figures-5j-l).

**Table-4:** Histopathology scoring of dog’s skin inflammation and its location after experimental inoculation with *S. aureus* and topical treatment with *A. vera* 20% and 40% and gentamicin 0.1% ointments.

Group	Untreated			*A. vera* 20% ointment			*A. vera* 40% ointment			Gentamicin 0.1% ointment		
			
	Epid	Der	S/C	Epid	Der	S/C	Epid	Der	S/C	Epid	Der	S/C
Zero day	+++	+++	+++	+++	+++	+++	+++	+++	+++	+++	+++	+++
3^rd^ DPT	+++	+++	+++	++	+++	+++	++	++	++	++	+++	+++
7^th^ DPT	+++	+++	+++	++	++	+++	+	+	++	++	++	+++
10^th^ DPT	++	+++	+++	+	+	++	–	+	+	+	+	++
14^th^ DPT	++	++	++	–	–	+	–	–	–	–	+	+

DPT=Days post pyoderma treatment, Epid=Epidermis, Der=Dermis, S/C=Subcutaneous tissues including fat and blood vessels. Scoring of histopathology (edema, necrosis, mononuclear cell infiltration and folliculitis); –: None; +: Mild <25%; ++: Moderate <50%; +++: Severe >50% of examined sections. *A. vera=Aloe vera*, *S. aureus=Staphylococcus aureus*

## Discussion

In the present study, the indicators of liver function (i.e., AST and ALT levels) and kidney function (e.g., BUN and creatinine) were higher in dogs experimentally infected with pyoderma; our results are similar to those reported for dogs with pyoderma and dermatophytosis [20] but disagree with the results of other studies [21,22]. The increased concentrations of BUN and creatinine may be due to dehydration caused by the hemorrhagic conditions revealed from pyoderma lesions, whereas the increased levels of AST and ALT may be related to increased levels of inflammatory cytokines, as reported in a previous study by our group [13]. Serum glucose concentrations were lower in the experimentally infected dogs compared with those of negative control dogs; these findings are in line with those previously documented in dogs [21]. Biochemical profiles revealed in the positive control group, making our results generally consistent with those of a previous study of dogs with atopic dermatitis [23]. Zinc plays a significant role in preserving lipid membranes against oxidation; thus, low zinc concentrations may act as a potential mechanism against reactive oxygen species production [24].

In this study, pyoderma appeared 3 days after inoculation; this differs from other studies, which describe the appearance of lesions 24 h after inoculation [25]. This variation may be due to differences with respect to the inoculated strain or the dogs’ age and breed. In terms of depth, canine pyoderma is divided into superficial bacterial folliculitis and deep pyoderma, including subcutaneous tissue, fat, and blood vessels [7,9,10]. Interestingly, both types of pyoderma were observed, with inflammation in hair follicles and deep in the subcutaneous tissue, causing cellulitis and panniculitis. Deep pyoderma has been reported to be more serious than superficial pyoderma [9].

Systemic antimicrobial treatment of pyoderma induces multidrug resistance. In addition, in some countries, the use of some antibiotics is limited in pets [9,10]. As such, topical treatment may be the most appropriate method for treating pyoderma in dogs [10]. *A. vera* is a natural plant rich in anthraquinones, polysaccharides, and pyrocatechol, a hydroxylated phenol. Anthraquinones have a similar action as tetracycline: The inhibition of bacterial protein synthesis [26]. Polysaccharides stimulate leukocyte phagocytic activity to kill bacteria [27]. Pyrocatechol has a toxic effect on microorganisms [28]. Through these components and others, as seen in this study, the topical application of *A. vera* ointment successfully induces skin healing and resolves the inflammatory changes caused by *S. aureus* inoculation, as confirmed by histopathology. In addition, *A. vera* ointment was able to treat deep pyoderma, which involved fat and blood vessels in the subcutaneous tissue. *A. vera* 40% ointment treated skin pyoderma more quickly than *A. vera* 20% and gentamicin 0.1% ointments; this may be explained by a dose-dependent concentration, as previous research has shown that high concentrations of several dilutions of *A. vera* extract can successfully inhibit *S. aureus* [29]. Moreover, *in vitro* assays previously confirmed the inhibitory effect of *A. vera* ointment on bacteria [30,31].

## Conclusion

Based on the biochemical and histopathological improvement of skin lesions, the topical application of *A. vera* 40% ointment may be a suitable herbal therapy against staphylococcal pyoderma in dogs. As such, *A. vera* 40% ointment is a suitable therapy, without the side effects associated with antibiotics, for use in the veterinary field.

## Authors’ Contributions

HYH: Designed the idea and experiment. AK, AA, AE, AhE, and HK: Executed the experiments and analyzed the samples. AK, AA, and AE: Interpreted the data and drafted the manuscript. AE: Histopathological part. AhE: Responsible for bacteriological share. AA and HK: Biochemical part. AK: Responsible for gel formation, statistical analysis and editing of the manuscript. All authors critically revised the manuscript for important intellectual content. HYH: Supervised the study. All authors read and approved the final manuscript.
